# When the neighbors are noisy: effect of social challenge in collateral pens of stressed animals

**DOI:** 10.3389/fvets.2024.1433628

**Published:** 2024-09-23

**Authors:** Raúl David Guevara, Sergi López-Vergé, Jose J. Pastor, Xavier Manteca, Gemma Tedo, Pol Llonch

**Affiliations:** ^1^AWEC Advisors S.L., Animal Welfare Education Centre (AWEC), Cerdanyola del Vallès, Spain; ^2^Department of Animal and Food Science, Universitat Autònoma de Barcelona, Barcelona, Spain; ^3^Animal Science Innovation Division, Lucta, Cerdanyola del Vallès, Spain

**Keywords:** social stress, pig welfare, pig behavior, piglets, emotional contagion, aggression

## Abstract

Regrouping practices are frequent in pig production, altering hierarchy and triggering aggressive behaviors. The present study aimed to investigate the physiological responses of piglets to an experimental model designed to induce stress through systematic social mixing in two trials. In Trial A, a total of 144 crossbred piglets (25 days postweaning) housed in one room within 36 pens (four piglets/pen) were used and randomly assigned to either a control group (piglets maintained in their pen, Ctrl-A) or a social challenge group (piglets mixed, SC-A). In Trial B, the same number of animals (33 days postweaning) and crossbreed line was used, and each piglet was assigned either to a control group (Ctrl-B) or a social challenge group (SC-B) in two independent rooms (rooms Ctrl and SC, 12 pens/ room, six piglets/pen). The social challenge consisted of daily moves of three out of four pen mates and five out of six pen mates, for Trials A and B, respectively. In the Ctrl groups, all piglets stayed in their original pen. Before the 1st mixing day and at the end of the 3rd mixing day, saliva (cortisol concentration) and blood (cortisol concentration changes, hemogram, and immunologic activation) samples were collected from two random piglets per pen. Skin lesion scores of all piglets were also recorded on the front, middle, and rear body regions. In Trial A, the total skin lesions score was higher in the SC-A group compared to the Ctrl-A group after the social challenge (0.53 vs. 0.17; *p* < 0.05), but an unexpected increase between sampling days in the Ctrl-A piglets (0.06 vs. 0.17; p < 0.05) was also recorded, suggesting that Ctrl-A pigs showed similar aggressivity levels to the SC-A group. Hematological parameters hemoglobin, red blood cell counts, and leukocyte counts present similar changes in both treatment groups after the social challenge. Contrarily, in Trial B, the lesion score only increased in the piglets in room SC (0.08 vs. 0.34; *p* < 0.05). Results suggest that stable groups may show aggressive behaviors if they are in the same room with socially challenged pigs. Thus, the physical separation of treatment groups in social stress studies is recommended.

## 1 Introduction

Social stress in pigs is often caused by common management practices (cross-fostering during lactation, litters’ mixing at weaning, pens’ mixtures at nursery and fattening, and transportation, among other events) that involve regrouping with unfamiliar individuals (1–3). During these mixing events, the established social order among pigs may be disrupted, leading to aggressive behaviors like fights, bites, intimidation, and competition for resources (4, 5). Pigs exhibit various behavioral responses when dealing with violent interactions during social mixing. These actions can be offensive, such as head-to-head clashes, head knocks, biting, chasing, and intimidating through grunts and threatening postures (6–8). Alternatively, responsive actions can be defensive, involving freezing in place or attempting to escape from confrontations (4). However, constant high levels of aggression can compromise the welfare of pigs due to social stress. Social stress can increase cortisol levels (8–10), acute phase protein concentrations (11, 12), immune activation (13, 14), and other hematological parameters (9, 13). On top of that, social stress can negatively affect animal performance and the physiological condition of pigs (13, 15).

Pig production stakeholders have been interested in the mitigation of agonistic behaviors (e.g., tail biting, fighting, intimidating, among others) as it affects the profitability of the production system (16) and raises social concerns about the production practices (17). Plenty of literature has already reported the negative impacts of agonistic behaviors on pigs but the methods to predict and control aggressive outbreaks remain unclear (5, 18, 19). Thus, pig production stakeholders are interested in understanding how negative behaviors disseminate among a group of animals, to develop and apply strategies (e.g., nutritional, husbandry, or management) to reduce the incidence of such negative behaviors.

The present study aimed to investigate the impact of social stress during mixing events and the occurrence of non-socially mixed piglets. A better understanding of negative behaviors dissemination might help to develop approaches to mitigate social stress in pigs.

## 2 Materials and methods

### 2.1 Animals, housing, and diets

All procedures were approved by the Laboratory Animal Care Advisory Committee of the Faculty of Veterinary Sciences of the Autonomous University of Barcelona (CEEAH-5754-5755-CEEA-UAB). Two independent trials were performed: (i) Trial A took place at the Institute of Agrifood Research and Technology (IRTA) experimental farm, Mas Bové (Constantí, Tarragona, Spain); and (ii) Trial B was carried out at the LUCTA^®^ Swine Experimental Unit transition farm, “El Castell” (Sant Aniol de Finestres, Girona, Spain). In both trials, piglets from 20 sows were selected (piglets over 4 kg body weight) to be moved to a nursery site at weaning. In trial A, this occurred approximately on day 26, and in trial B, it happened approximately on day 29. At the nursery site, the piglets were distributed according to sex and body weight, and all animals were identified with ear tags.

For Trial A, a total of 144 25-days postweaning (p.w.) crossbred [(Largewhite × Landrace) × Pietrain] piglets (BW 11.90 kg ± 0.79 kg) were housed in one room and distributed into 36 pens according to sex (2 female and 2 male/pen). Each individual pen (4.42 m^2^; 2 × 2.21 m) was equipped with a hopper feeder featuring four eating spaces, and a drinker.

The same number of animals (BW 14.42 kg ± 0.37 kg, 33 days p.w.) and crossbreed line were used in Trial B. However, pigs were housed in two separate rooms, each containing 12 pens (2.7 m^2^; 2.2 × 1.3 m), allocating six piglets per pen, maintaining the same proportion of males and females in each pen (3 female and 3 male/pen). Each pen had two drinkers covered with a mobile metal lid to prevent external contamination (feces and urine) and minimize water waste. Pens had one feeder per pen, with 4 separations per feeder, and were also be covered with a metal lid.

In both trials, all pens were equipped with completely slatted plastic floors; each pen had plastic chewable toys (plastic balls with chains attached to the wall); and feed and water were offered *ad libitum* throughout the experimental period (see Table 1). All diets were formulated to meet or exceed (20) nutrient requirements.

**Table 1:** Nutrient composition of the diets supplied during the starter phases of the trials (from day 21 to day 41 p.w.).

Nutrient	Trial A	Trial B
Digestible energy, Mcal/kg	3.28	3.45
Crude Protein, %	18.90	17.18
Calcium, %	0.70	0.73
Total phosphorus, %	0.68	0.58
Lysine, %	1.28	1.20
Methionine, %	0.48	0.48
Threonine, %	0.83	0.85
Tryptophan, %	0.26	0.23

### 2.2 Study design–social challenge

In both trials piglets were randomized to one of the two treatment groups: (i) control (Ctrl); and (ii) socially challenged (SC). The social challenge consisted of the daily relocation of three out of four pen mates and five out of six pen mates for Trials A and B, respectively. Relocated piglets were moved to pens with other piglets of similar body weight to maintain groups with similar average body weights. Meanwhile, in the Ctrl pens, all piglets remained in their original pens. The process of social mixing was conducted for three consecutive days, starting at 8:00 a.m. During the 3 days of social challenge, systematic regrouping events were done at 8:00 am before food was placed on the feeders. On the fourth day of the social challenge period, all animals returned to their original pens at 8:00 a.m. for sampling (saliva and blood) and skin lesion score measurements.

In Trail A, both Ctrl and SC pens were housed in the same experimental room, whereas in Trial B, Ctrl, and SC pens were housed in two separate rooms (i.e., one room of Ctrl pens and one room of SC pens).

### 2.3 Sampling and measurements

#### 2.3.1 Lesion score

Lesion score recording was meant to measure piglets’ aggressiveness during the social mixing events.

**Figure 1:**
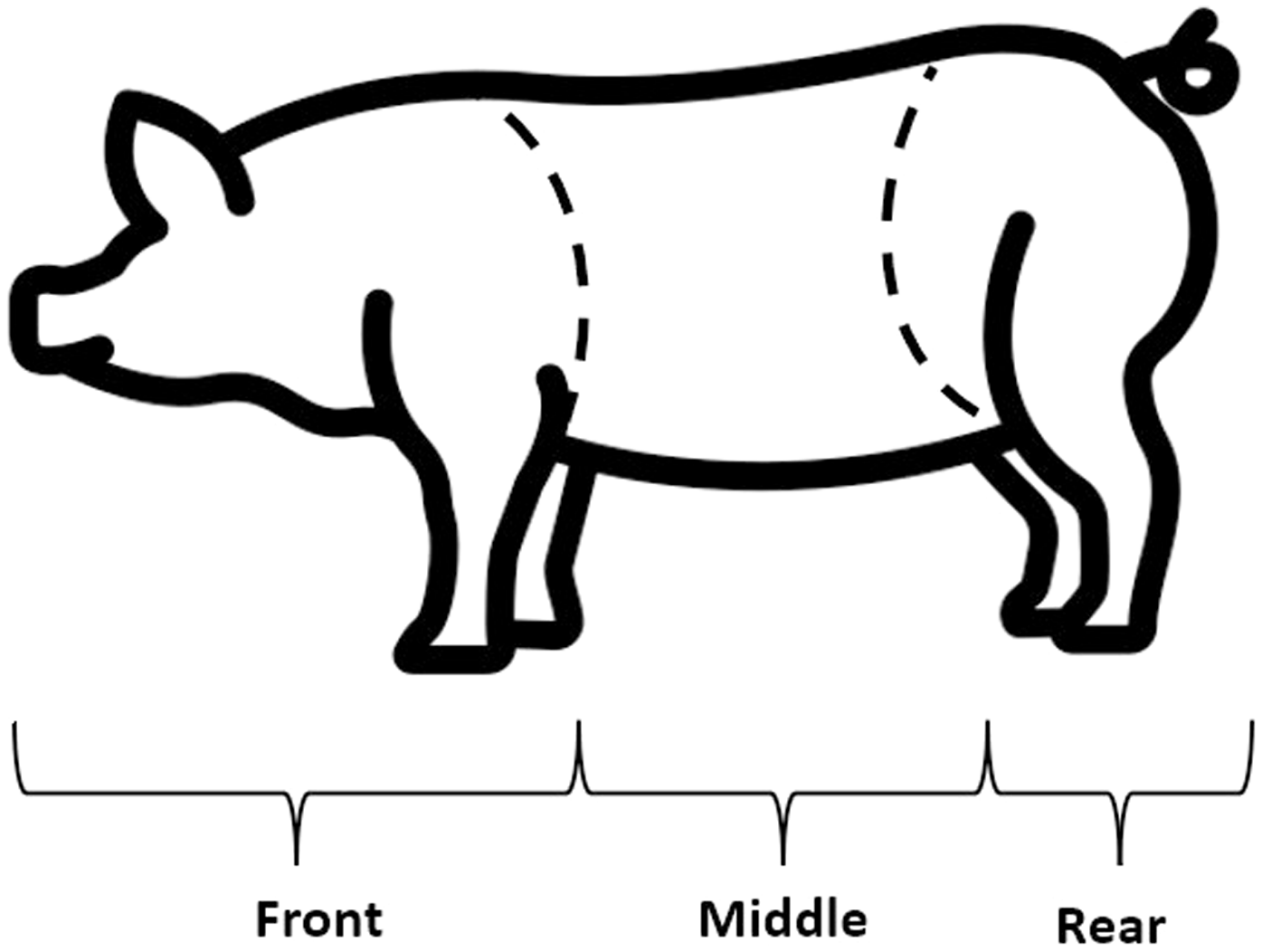
Pig body regions assessed through the skin lesion score (21).

On trial A, lesion scores were recorded twice, on the first day and on the last day of the social challenge (days 25, 28 p.w.). Whereas in trial B, the lesion score was recorded thrice, each morning of the social challenge period before pen mixtures (days 33, 34, and 35 p.w.). More skin lesion observations were done in trial B to refine aggressions detection during the challenge period, which was an improvement of the observation protocol from trial A. The lesion score assessment protocol was based on Turner et al. (21), counting the number of lesions on each of the three areas of the body: front, middle and rear, to assign a score from 0 (<6 lesions), 1 (6–15 lesions), or 2 (>15 lesions) (Figure 1). The lesion score aimed to measure the aggressivity of the pigs during the social challenge period.

#### 2.3.2 Blood, and saliva samples collection

Blood samples were collected to detect hematological changes triggered by the social mixing. Also, plasmatic and salivary cortisol concentration was measured to monitor piglets stress response.

Piglets were fasted overnight before the sampling to reduce variability in the markers (hematological parameters). Blood samples were collected from the vena *cava cranialis* using a vacutainer into pre-labeled tubes with EDTA anticoagulant. The blood tubes were immediately centrifuged (1200 *g* × 10 min). After centrifugation, supernatant was collected, frozen immediately in dry ice and stored (−80°C) until analysis (22).

Saliva samples were collected using saliva collection tubes containing a sponge (Sarstedt, Aktiengesellschaft & Co., Nümbrecht, Germany) each sampling day at the same time (±30 min) to avoid variations due to physiological circadian rhythm. A sponge clipped to a hemostatic clamp was playfully presented to the pigs to encourage them to chew it. Pigs were allowed to chew for approximately 1 min, and subsequently, the samples underwent a centrifugation cycle at 3000 *g* for 10 min at room temperature. Immediately after centrifugation, the samples were rapidly frozen using dry ice and stored at −80°C until analysis (23).

### 2.4 Sample analyses

Samples were analyzed for stress activation: cortisol concentration, hematology, hemoglobin, red blood cell counts (RBC), medium corpuscular volume (MCV), mean corpuscular hemoglobin (MCH), and mean corpuscular hemoglobin concentration (MCHC). Cortisol concentration measurements were done aiming to detect a stress response in the animals after the social challenge period. Platelet counts, leukocyte counts, eosinophil counts, basophil counts, lymphocyte counts, monocyte counts, and segmented neutrophil counts were used to evaluate the activation of the immune system before and after the social challenge.

Blood samples were analyzed in a commercial lab (Echevarne, Barcelona, Spain).

Saliva samples were analyzed for cortisol concentration through an automated chemiluminescence immunoassay (Immulite 1000, Siemens Medical Solutions Diagnostics), previously validated for its use in pigs by Escribano et al. (24).

### 2.5 Statistical analyses

All statistical analyses were performed with the statistical package SAS (version 9.4, SAS Institute Inc., Cary, NC, USA). PROC GLIMMIX was used to assess the lesion score changes between pre pre-social challenge record and post-challenge record in both trials. PROC MIXED with piglet as a random effect and sampling day and treatment as a fixed effect, and adjustment by Tuckey was used to analyze the hematology parameters in the trials. Salivary stress markers activity was performed by PROC GLIMMIX without any adjustment. The main effects and the interaction between them were compared by LSMEANS. Significant differences were declared at *p* ≤ 0.05 whereas near-significant trends were considered at 0.05 < *p* ≤ 0.10. Results are presented as mean values with their standard error from the mean (Mean ± SEM).

## 3 Results

### 3.1 Lesion score

**Figure 2:**
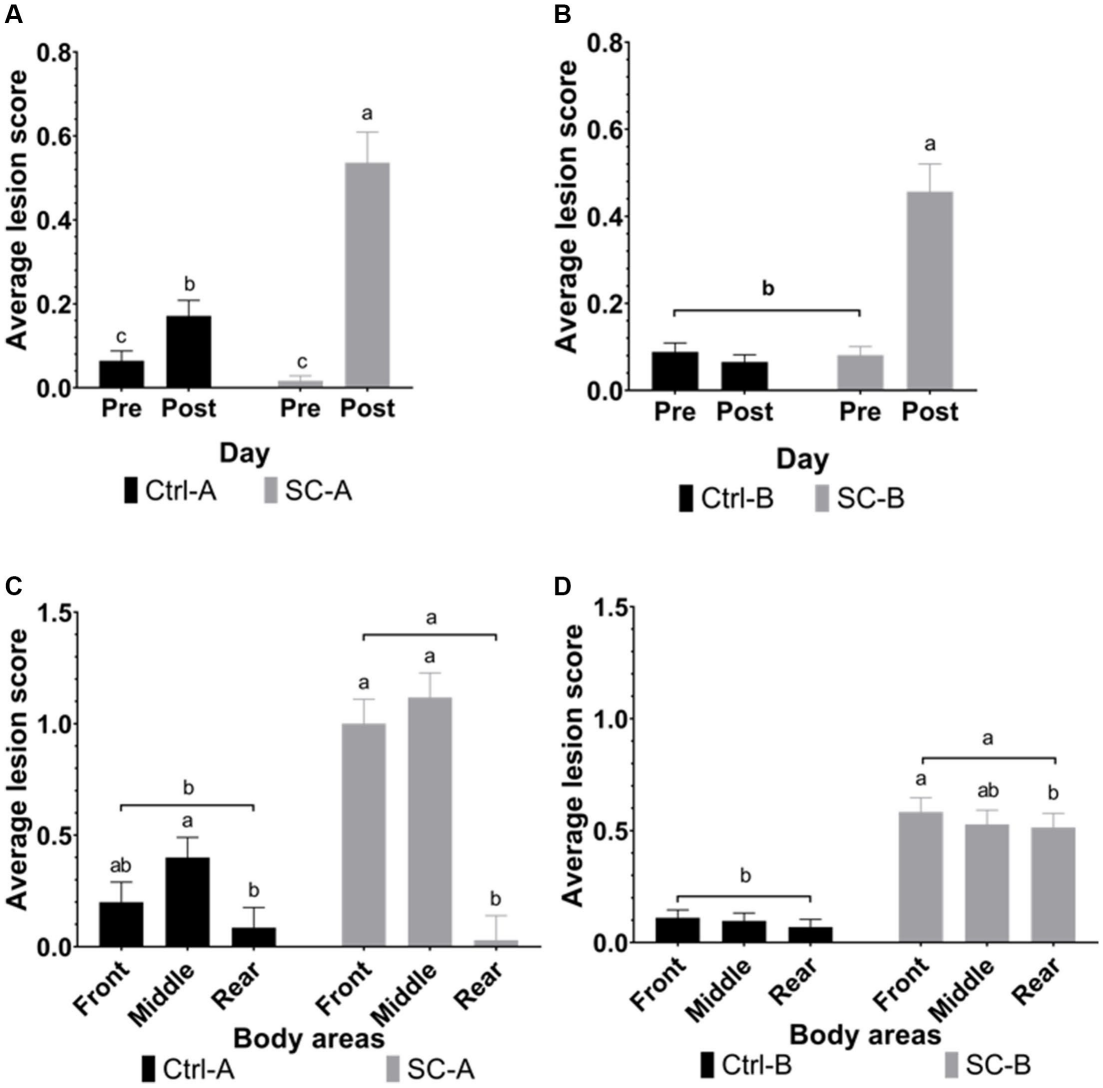
Lesion scores were grouped by treatment groups on days pre- and post-social mixing. **(A)** Trial A: all animals in the same experimental room at 25 days after weaning (pre-social challenge) and at 28 days after weaning (post-social challenge). **(B)** Trial B: animals with each treatment group allocated in separate rooms at 33 days after weaning (pre-social challenge) and 36 days after weaning (post-social challenge). **(C)** Body lesions distribution in trial A at 28 days after weaning (post-social challenge). **(D)** Body lesions distribution in trial B at 33 days after weaning (post-social challenge). Different lowercase letters indicate significant differences (*p* < 0.05) whereas near-significant trends were considered at 0.05 < *p* ≤ 0.10 and represented with capital letters.

Figure 2 illustrates the lesion scores observed in both trials. No differences were found between Ctrl-A and SC-A (day 25; *p* = 0.4645) or between Ctrl-B and SC-B (day 33; *p* = 0.6257) on their initial lesion scores. After the social mixing, SC-A had a significant increase in the lesion score, relative to the pre-social mixing measurement (*p* < 0.0001). Ctrl-A piglets also displayed an increase in the lesion score, despite not being directly exposed to social mixing (*p* = 0.0127), although it was reduced in lower magnitude compared with the SC-A. Regarding the distribution of body lesions, both Ctrl-A (*p* = 0.0101) and SC-A (*p* < 0.0001) treatments show a higher concentration of lesions in the front and middle areas relative to the rear body area.

SC-B pigs presented a higher lesion score after the social mixing than the measurement previously to the mixing (*p* < 0.0001), but Ctrl-B pigs did not increase their lesion score after the social mixing period (*p* = 0.3379). Concerning the distribution of the lesions in the body areas, SC-B piglets presented a higher concentration of body lesions in the front (*p* = 0.0158) relative to the rear body area but not different from the middle body area (*p* = 0.0677). SC-B middle area lesion score was similar to the rear body area (*p* = 0.8412). On the other hand, Ctrl-B pigs maintained their body lesion score in the different body areas after the social mixing period (*p* = 0.0140).

### 3.2 Blood markers

#### 3.2.1 Hematological parameters

Hematological parameters summary for trials A and B are summarized in Tables 2, 3, respectively.

**Table 2:** Hematological parameters from treatment groups Ctrl-A and SC-A before (Pre) and after (Post) social challenge period (from day 25 post-weaning until day 28) on trial A.

	Ctrl-A				SC-A			
Parameter	Pre	Post	SEM	*p*-value	Pre	Post	SEM	*p*-value
Hematocrit, %	34.39	35.34	2.323	0.0672	33.21	32.36	2.574	0.0998
Hemoglobin, g/dL	**10.99**	**10.61**	**0.618**	**0.0060**	**10.51**	**9.71**	**0.842**	**<0.0001**
Red blood cells, 10^12^/L	**6.36**	**6.18**	**0.552**	**0.0299**	**6.01**	**5.63**	**0.640**	**<0.0001**
MCV, fL	**54.34**	**57.44**	**4.787**	**<0.0001**	**55.56**	**57.75**	**4.098**	**0.0005**
MCH, pg	17.39	17.25	1.475	0.0901	**17.56**	**17.30**	**0.872**	**0.0019**
MCHC, g/dL	**31.97**	**30.05**	**1.128**	**<0.0001**	**31.68**	**29.99**	**1.488**	**<0.0001**
Platelets, 10^12^/L	0.56	0.63	0.132	0.0515	0.52	0.53	0.180	0.8265
Leukocytes, 10^12^/L	**0.02**	**0.02**	**0.007**	**0.0098**	**0.02**	**0.02**	**0.006**	**0.0030**
Eosinophils, /μL	319.13	274.78	133.260	0.5410	361.69	338.50	219.351	0.7690
Basophils, /μL	238.94	214.83	75.470	0.2228	245.88	209.50	60.580	0.0692
Lymphocytes, /μL	14021.93	12946.00	4247.790	0.0595	**15258.59**	**12256.82**	**3831.760**	**0.0004**
Monocytes, 10^12^/L	0.01	0.01	0.002	0.3928	**0.01**	**0.01**	**0.002**	**0.0008**
Neutrophils, 10^12^/L	0.01	0.01	0.002	0.3928	0.01	0.01	0.002	0.6884
Neutrophils/Lymphocytes ratio	0.46	0.43	0.213	0.7572	0.38	0.49	0.218	0.0681

Bold numbers indicate significant differences (*p*<0.05) between measurements taken pre- and post- social challenge period.

**Table 3:** Hematological parameters from treatment groups Ctrl-A and SC-A before (Pre) and after (Post) social challenge period (from day 33 post-weaning until day 36) on trial B.

	Ctrl-B				SC-B			
Parameter	Pre	Post	SEM	*p*-value	Pre	Post	SEM	*p*-value
Hematocrit, %	**29.95**	**32.33**	**2.962**	**0.0013**	29.93	31.53	2.833	0.0602
Hemoglobin, g/dL	9.40	9.46	0.908	0.7513	9.61	9.40	0.724	0.1703
Red blood cells, 10^12^/L	5.38	5.39	0.492	0.9081	5.64	5.54	0.504	0.1514
MCV, fL	**55.64**	**60.02**	**1.755**	**<0.0001**	**53.14**	**56.94**	**2.223**	**<0.0001**
MCH, pg	17.47	17.57	0.695	0.2044	17.08	16.99	0.789	0.8704
MCHC, g/dL	**31.40**	**29.27**	**0.764**	**<0.0001**	**32.13**	**29.85**	**1.144**	**<0.0001**
Platelets, 10^12^/L	0.54	0.59	0.105	0.4874	0.49	0.56	0.156	0.1291
Leukocytes, 10^12^/L	0.02	0.02	0.006	0.7614	0.02	0.02	0.007	0.0663
Eosinophils, /μL	249.79	241.33	315.898	0.9297	142.80	295.19	194.858	0.1308
Basophils, /μL	155.65	88.26	148.064	0.2159	105.52	75.95	148.302	0.5898
Lymphocytes, /μL	10188.78	11628.56	3547.630	0.2027	**7441.83**	**10115.56**	**3572.190**	**0.0262**
Monocytes, 10^12^/L	0.00	0.00	0.001	0.0883	0.00	0.00	0.006	0.2921
Neutrophils, 10^12^/L	0.01	0.01	0.004	0.1224	0.01	0.00	0.006	0.9034
Neutrophils/Lymphocytes ratio	0.73	0.46	0.447	0.2112	**1.27**	**0.80**	**1.177**	**0.0430**

Bold numbers indicate significant differences (*p*<0.05) between measurements taken pre- and post- social challenge period.

In trial A, Ctrl-A eosinophils, basophils, and neutrophils counts did not present differences after the social mixing. SC-A platelet, eosinophils, and neutrophils counts were not different between pre- and post-social challenge. The Ctrl-A ratio of neutrophils/lymphocytes was not different pre- and post-challenge, but the SC-A neutrophils/lymphocytes ratio presented a trend to increase after the challenge period (29.79%). On the other hand, hemoglobin concentration (3.48%), RBC count (2.79%), MCHC (6.00%), leukocytes (13.61%), and monocytes (29.37%) counts were reduced after the social challenge period in the Ctrl-A piglets. Similarly, SC-A piglets had significant reductions in hemoglobin concentration (7.66%), RBC count (6.36%), MCH concentration (1.48%), MCHC (5.33%), leukocytes (17.14%), lymphocytes (19.67%), and monocytes (21.39%) counts after the social mixing. MCV increased after the social mixing in both Ctrl-A (5.71%) and SC-A (3.94%) groups. Ctrl-A MCH concentration (0.77%) and lymphocyte counts (7.67%) tended to decrease, and hematocrit percentage (2.76%) and platelet counts (12.59%) tended to increase after the social challenge in the Ctrl-A group. The SC-A hematocrit percentage (2.55%) and basophil count (14.79%) tended to be reduced after the social challenge period. When comparing treatment groups and the interaction of the treatment*day factors in terms of the different hematological parameters, no significant differences were found (*p* > 0.05).

On trial B, no significant differences between the pre- and post-social challenge were detected on Ctrl-B piglets’ hemoglobin and MCH concentrations, RBC, platelets, leukocytes, eosinophils, basophils, lymphocytes, and neutrophils counts, and on SC-B piglets’ hemoglobin and MCH concentrations, RCB, platelets, eosinophils, basophils, monocytes and neutrophils counts. The Ctrl-B- neutrophils/lymphocyte ratio did not differ between pre- and post-challenge period measurements. Contrarily, Ctrl-B piglets’ hematocrit percentage (7.92%) and MCV (7.87%) were increased after the social challenge period, but Ctrl-B MCHC decreased after the challenge period (6.78%). SC-B pigs’ MCV concentration (7.17%) and lymphocyte count (35.92%) increased after the social mixing, although SC-B MCHC was reduced after the social challenge (7.06%). Monocyte count tended to increase on Ctrl-B piglets after the challenge period (3.84%), and hematocrit concentration (5.36%) and leukocyte count (17.75%) tended to increase after the challenge on the SC-B piglets. The SC-B neutrophil/lymphocyte ratio decreased significantly (37.04%) after the social challenge. Similarly to trial A, there were no significant differences observed between treatment groups or the interaction treatment*day in the various hematological parameters (*p* > 0.05).

#### 3.2.2 Plasmatic cortisol

**Figure 3:**
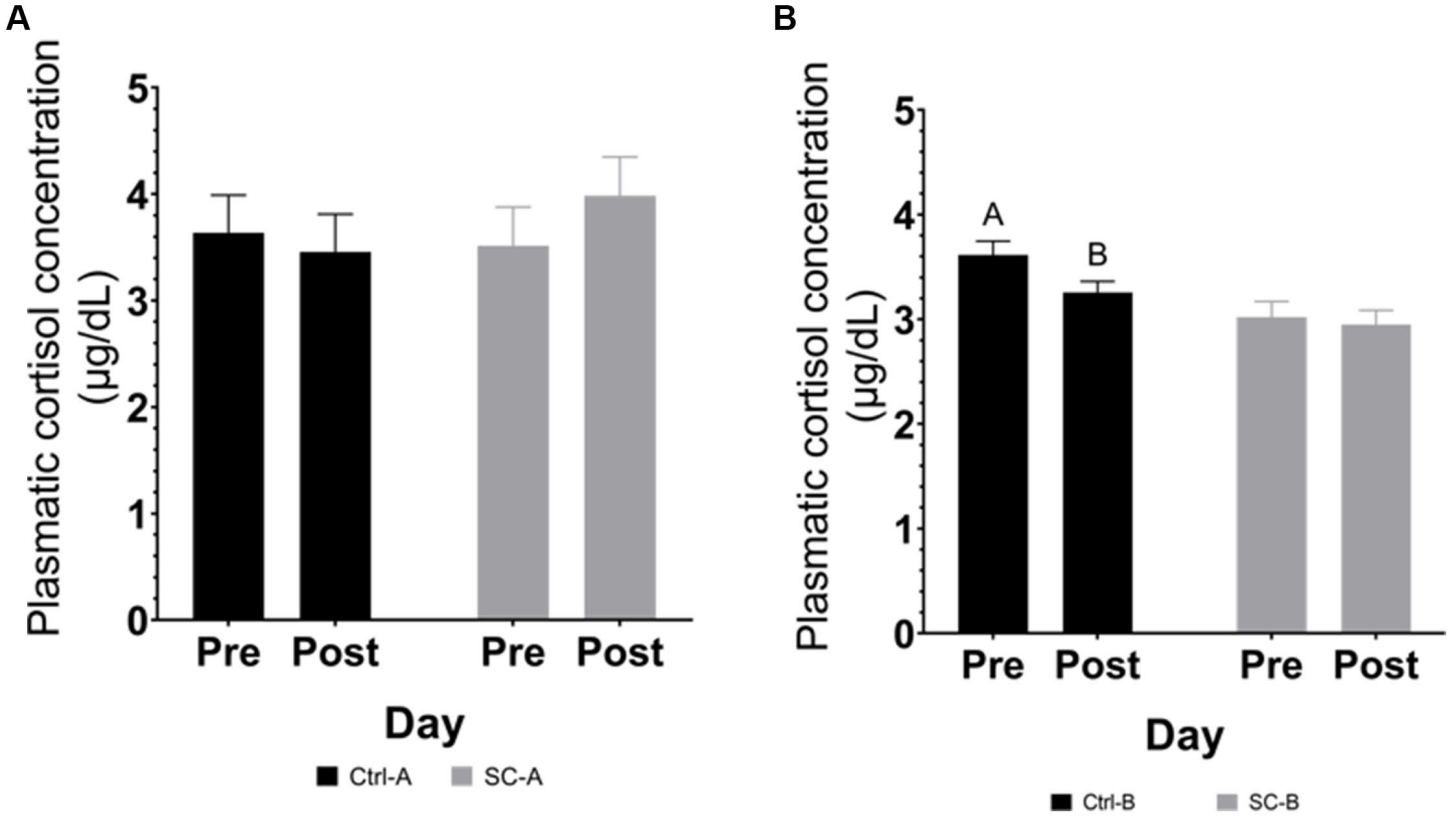
Plasmatic cortisol concentration by treatment groups on days pre- and post-social mixing. **(A)** Trial A: all animals in the same experimental room at 25 days after weaning (pre-social challenge) and at 28 days after weaning (post-social challenge). **(B)** Trial B: animals with different treatments in different rooms at 33 days after weaning (pre-social challenge) and 36 days after weaning (post-social challenge). Different lowercase letters indicate significant differences (*p* < 0.05) whereas near-significant trends were considered at 0.05 < *p* ≤ 0.10 and represented with capital letters.

Plasmatic cortisol in trials A & B are depicted in Figure 3. In trial A, no significant differences in plasmatic cortisol were detected after the social challenge (between day 25 and day 28 after weaning) in any treatment group (Ctr-A *p* = 0.7202; SC-A *p* = 0.3623). Plasmatic cortisol concentration in the Ctrl-B had a declining trend (*p* = 0.0711) after the social challenge period (between day 33 and day 36 after weaning). On the other hand, the plasmatic cortisol concentration of SC-B piglets was not different after the social challenge (*p* = 0.7187).

#### 3.2.3 Salivary cortisol

**Figure 4:**
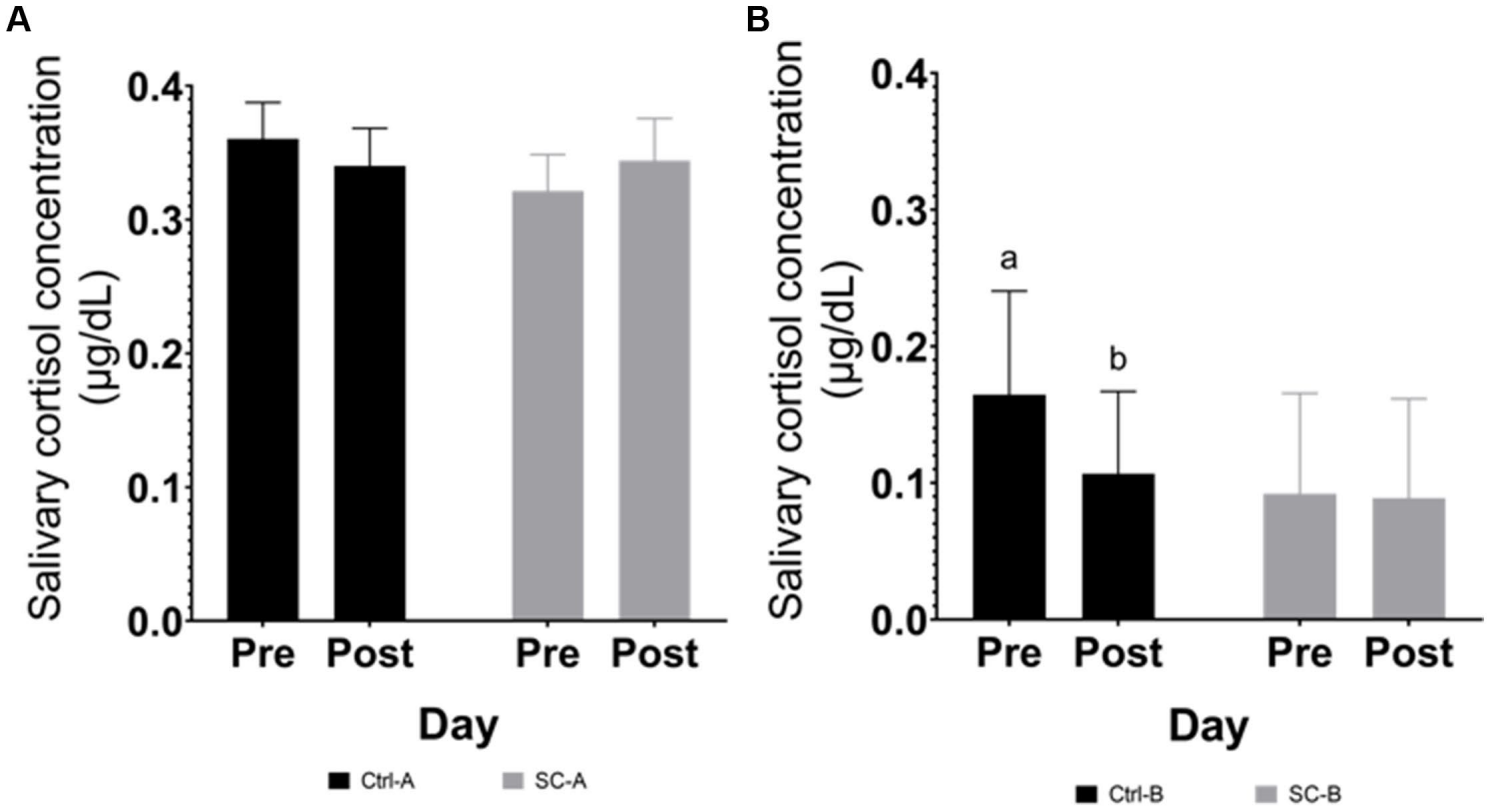
Salivary cortisol concentration by treatment groups on days pre- and post-social mixing. **(A)** Trial A: all animals in the same experimental room at 25 days after weaning (pre-social challenge) and at 28 days after weaning (post-social challenge). **(B)** Trial B: animals with different treatments in different rooms at 33 days after weaning (pre-social challenge) and 36 days after weaning (post-social challenge). Different lowercase letters indicate significant differences (*p* < 0.05) whereas near-significant trends were considered at 0.05 < *p* ≤ 0.10 and represented with capital letters.

Cortisol concentration in saliva is represented in Figure 4. In Trial A no significant differences were detected in any of the tested conditions, neither treatment nor date (between day 25 and day 28 after weaning). In Trial B, Ctrl-B piglets displayed higher cortisol concentration than SC-B piglets (*p* = 0.0187) before the social stress (day 33 after weaning). After the stress period (day 36 after weaning), Ctrl-B showed a reduction in salivary cortisol (*p* = 0.0394) while no salivary cortisol changes were detected in the SC-B group (*p* = 0.9480).

## 4 Discussion

The spread of agonistic behaviors among pigs may trigger social stress (21). Social stress in pig production is considered a major animal welfare issue (14). Therefore, a deep understanding of the factors that can trigger and spread aggression among pigs is important for production stakeholders to minimize stress and improve animal welfare. The goal of the current research was to study the appearance and effects of agonistic behaviors triggered by systematic social mixing procedures in piglets. This knowledge will help pig stakeholders develop strategies to mitigate the impacts of social stress on pigs.

In Trial A, both Ctrl-A and SC-A treatment groups were randomly placed in the same experimental room. In contrast in Trial B, Ctrl-B and SC-B piglets were placed in separate experimental rooms. As anticipated, piglets exposed to social mixing in both trials (SC-A and SC-B) exhibited significant increases in body lesion scores. This outcome aligns with findings from other studies that also investigated the impacts of social mixing (21, 25, 26). Surprisingly, Ctrl-A piglets also displayed a notable increase in body lesion scores, although to a lesser degree compared to the pronounced increase observed in SC-A piglets following social mixing. It is worth noting that this increase occurred even in the absence of deliberate exposure to the social challenge. Furthermore, the observed body lesions in both Ctrl-A and SC-A groups were primarily concentrated in the front and middle areas of their bodies. Several authors (21, 27–29) reported that lesions accumulation in the front and middle areas are related to fights and confrontations, while body lesions in the rear part of the body of the pig might be related with insufficient access to the feeder or the drinker, respectively. Additionally, the change in hematological parameters was comparable in both Ctrl-A and SC-A pigs after social mixing. Therefore, it is possible to assume that Ctrl-A piglets engage in agonistic interactions and confrontations without experiencing a social challenge. Contrarily, in trial B, where the treatment groups were physically separated, Ctrl-B lesion scores and hematological parameters were unaffected during the social mixture period, while the SC-B piglets had a significantly higher lesion score.

Regarding the hematological parameters measured, pre- and post-social challenge results fell within the reference intervals (30). Hematological parameters are particularly sensitive to the impacts of stress, influencing fundamental functions like growth, maintenance, and immune activities (31–33). Hematocrit percentage, hemoglobin concentration, and red blood cell counts have been shown to have connections with pig growth and voluntary feed intake (34). In both Ctrl-A and SC-A groups, there was a similar pattern in hemoglobin concentration and red blood cell counts after the social challenge. These indicators displayed decreased levels, likely influenced by the stress experienced by the piglets (35, 36). Parameters linked to the morphology of red blood cells, such as MCV and MCHC, were similarly influenced in both Ctrl-A and SC-A piglets. This similarity suggests that social stress was induced in SC-A pigs, whereas Ctrl-A pigs might experience a stress response triggered by the higher activity caused by the pen neighbor’s noise or the auditory cues of aggression from other pigs in the room.

Concerning immune activity parameters, the process of social mixing led to a decrease in the activity of leukocytes, lymphocytes, and monocytes in both Ctrl-A and SC-A piglets. Acute stress can disrupt the typical concentration of these indicators, as cortisol affects the organism’s response (31, 33). Meanwhile, in Trial B, where both Ctrl-B and SC-B pigs were physically separated, the immune activity of Ctrl-B pigs remained unaffected following the social challenge period. The only change observed was an increase in hematocrit percentage among Ctrl-B pigs during that timeframe, which could be attributed to their natural growth pattern as the piglets are in the growing stage (34). On the other hand, lymphocyte activity increased after the social mixing in the SC-B piglets. Thus, Ctrl-B piglets were not affected at the same level as Ctrl-A during the social mixing period, and SC-B piglets did show a milder response to social mixing compared to SC-A. Even if aggression levels were expected to be more severe in SC-B piglets, compared to SC-A, as older or heavier animals are expected to engage in more intense agonistic interactions (7, 37, 38), skin lesion score and hematological parameters were less severe relative to SC-A piglets. This milder physiological response of the SC-B piglets might be related to the fact that the piglets on trial B were older and heavier relative to trial A piglets, they might have been able to cope better with the physiological imbalance, which could have made them more resilient to the social challenge (39). The reductions of MCHC in both Ctrl and SC in both trials A and B might be related to the acute stress of the sampling protocol performed to obtain the blood samples (31).

In both trials, measurements of cortisol concentration in both blood and saliva were unable to identify changes following the social mixing period. This result might be attributed to the sampling schedule, potentially missing the peak of cortisol activity. Cortisol levels are expected to increase as a physiological response to the aggression arising from social mixing, including fights, confrontations, and intimidations (9, 40, 41). This reaction has been observed to occur within the initial hours (ranging from 40 min to 5 h) following the stress event (4, 42, 43). Moreover, pig confrontations resulting from social mixing have been documented to take place within the initial 90 min after mixing (4). In this study, saliva and blood sampling were conducted 24 h after the final social mixing, once the pigs had returned to their original pen. Post social mixing sampling point might have overlooked the peak cortisol concentration as cortisol concentration might be dropped after 24 h of stress peak (44). The initial intention of cortisol (blood and saliva) pre and post-social mixing was to minimize constant manipulation for the piglets that might add stress due to handling or operator presence, affecting the physiological responses of the pigs (45). Additionally, the purpose of the repeated social mixing protocol (spanning 3 consecutive days) was to escalate social stress. Nonetheless, the repetition of the social mixing event could lead to a decrease in the intensity of fights and confrontations (4), the intensity observed after the mixing period could potentially be the least among the 3 days of the social challenge. Cortisol or any other biomarker as a stress indicator is fragile and easily disrupted by several factors (44), some of which are the handling (invasive) procedures and the appropriate time to collect the measurement sample. Moreover, these traditional measurement methodologies are time-spot and might lose information (18), as occurred in the current study. As an alternative to traditional measurements, behavioral assessments (remote method) are emerging as a solution to detect abnormalities or a high incidence of agonistic behaviors (18, 46). Computer vision and machine learning have boosted the evolution of technology capable of detecting situations that can trigger stress and deteriorate the welfare of animals in production conditions. Gómez et al. (47), present computer vision technologies (i.e., cameras, accelerometers, and other PLF devices to record animal activity and aggressive interactions) externally or internally validated capable of detecting and measuring antagonistic activities among pigs.

Therefore, two hypotheses might be proposed based on the results obtained and the interpretation of the information collected (i.e., biomarkers and behavioral information). When pigs are exposed to loud noises, it can change their behavior, such as increasing their activity level and curiosity toward the source of the sound or causing them to freeze. Also, it can raise their heart rate (48). Therefore, during the social mixing process on a farm, the loud sounds (i.e., vocalizations, grunts, stepping sounds, sounds of the lids closing sounds, and sounds from piglets’ collisions against the pen walls) from the staff and the activity in the mixed pens could cause anxiety and fear in the Ctrl-A piglets, who were not part of the mixing process. This could increase the physical activity of the Ctrl-A piglets and the chance to invade other individuals’ spaces, making them more likely to engage in confrontations and display aggressive behaviors. According to a study by Talling et al. (48), loud noises can cause small behavioral responses such as freezing, curiosity, or higher physical activity. However, Talling et al. did not describe if such higher activity triggered by the noise could provoke intense behavioral responses like fighting or escaping.

Another more complex hypothesis to explain the unexpected display of aggressive behaviors in Ctrl-A piglets, even in the absence of direct exposure to social mixing, might be the phenomenon of emotional contagion. Emotional contagion has been described as “*The emotional state matching of a subject with an object*” (i.e., the adoption of emotions of others) (49). Emotional contagion is provoked when an individual perceives another individual’s emotions and mimics the behavioral and physiological states of the transmitter (50). Emotional contagion has been theorized as a communication strategy to share environmental information with members of the community (51, 52). This information and cues can affect the physiological status, and the behavior of the observer animal (49, 50). Emotional contagion can disseminate emotional valence (positive or negative), and arousal level (high and low) (53). In line with this, a potential explanation is that the fighting grunts and fear/pain vocalizations (auditive cues) of piglets engaged in confrontations during the social challenge could potentially make Ctrl-A piglets more anxious and predisposed to engaging in fights with their pen mates (54, 55). Emotional contagion seems conceivable when the behavior and physiological responses of the observer align with those of the stressed individual (50). This phenomenon can be conveyed through visual means (54) or auditive cues (56–59). Only a limited number of studies have focused on comprehending the factors that govern this behavioral mimicry mechanism. Nonetheless, research in other species (i.e., rodents, nonhuman primates, ruminants, dogs, birds, and fishes, among others), as mentioned earlier, has extensively explored this phenomenon (52). However, the current experimental design does not allow for the confirmation of the hypothesis of emotional contagion.

## 5 Conclusion

In this study, the social challenge used resulted in aggressive behaviors among piglets, provoking mild to acute social stress in both trials. The social stress response was observed in the mixed piglets due to changes in the physiological condition of the animals. This was evidenced by an increase in body lesions and alterations in hematological parameters. Conversely, piglets without social mixing events also engaged in confrontations with their pen mates, as indicated by an increased skin lesion score and similar patterns in hematological markers compared to mixed piglets when they were in the same experimental room. This might suggest a phenomenon of contagion among the experimental pens. When socially mixed piglets and non-mixed piglets were physically located in different rooms, no changes were detected in the non-socially mixed pigs as a result of aggressive behaviors from socially mixed piglets. Consequently, future research endeavors must acknowledge that treatment groups causing behavioral responses, such as general noise or pain-related or stress vocalizations, could potentially alter the behavior of the animals in different treatment groups.

Moreover, invasive and time spot monitoring methods did not completely detect the physiological responses triggered by the social challenge. It’s important to note that spot-time measurements and scan samples may miss critical information that holds key relevance for concluding studies and research projects. The development of remote, minimally invasive, and continuous measurement methods is of great importance for animal research. These methods enhance the accuracy of monitoring animal welfare without causing disruptions to the animals. Furthermore, to collect the most information triggered by social stress, measurements for aggressive behaviors and stress biomarkers are suggested to be collected within the following hours after the regrouping event.

## Data Availability

The raw data supporting the conclusions of this article will be made available by the authors, without undue reservation.
